# Knowledge of HPV and acceptability of HPV vaccine among women in western China: a cross-sectional survey

**DOI:** 10.1186/s12905-018-0619-8

**Published:** 2018-07-27

**Authors:** Junyong He, Lixia He

**Affiliations:** Health Management Center of West China Hospital of Sichuan University, No. 37 Guo Xue Xiang, Chengdu, Sichuan 610041 People’s Republic of China

**Keywords:** HPV, Cervical cancer, HPV knowledge, Awareness, HPV vaccine

## Abstract

**Background:**

Since most cervical cancer cases are caused by persistent high-risk human papillomavirus (HR-HPV) infection, knowledge of HPV among women is essential for the prevent of cervical cancer. This study was aimed to assess knowledge among women in western China about HPV and its association with cervical cancer, and to assess their acceptance of HPV vaccination.

**Methods:**

A sample of healthy women undergoing routine physical examinations in the Health Management Center of West China Hospital, Sichuan University between January and December 2014 completed a questionnaire.

**Results:**

A total of 1300 questionnaires were distributed, and 1109 were completed and analyzed. Only 28.85% of respondents (*n* = 320) had heard of HPV; among this subgroup, only half (53.44%) knew that it causes cervical cancer, only 26 (8.13%) correctly answered all questions about HPV. Multivariate analysis showed that respondents who had heard of HPV were more likely than other respondents to have a family history of any cancer, to undergo regular Pap tests and to have completed at least secondary education. Half of all respondents (51.22%) reported that they would be willing to be vaccinated against HPV.

**Conclusion:**

Although most women in western China lack basic knowledge about HPV, at least half are willing to take the HPV vaccine. Public health efforts to educate the public about HPV and its connection to cervical cancer should be strengthened and expanded.

**Electronic supplementary material:**

The online version of this article (10.1186/s12905-018-0619-8) contains supplementary material, which is available to authorized users.

## Backgroud

Cervical cancer is the fourth most common cancer among women worldwide, with an estimated 528, 000 new cases and 266, 000 deaths in 2012 [1]. In China, 130,000 new cases of cervical cancer are diagnosed annually, cervical cancer is the second most common cancer in Chinese women aged 15–45 [2], despite the fact that the Pap smear test is well integrated into the Chinese healthcare system and widely accepted by women.

Most cervical cancer cases are caused by persistent high-risk human papillomavirus (HR-HPV) infection [3–5]. HPV infection is sexually transmitted, and most HPV infections are temporary and asymptomatic, and cleared by body’s immune response without treatment, co-factors to the suspected viruses are necessary [6]. Current clinical guidelines recommend screening women aged 30 and older using a Deoxyribonucleic acid (DNA)-based HPV test. This test is also recommended for women of any age with abnormal cytology results [7]. Therefore, promoting awareness of HPV and its link to cervical cancer among sexually active women is essential for the clinical practice of HPV-DNA test in cervical cancer screening.

Two HPV vaccinations have proven effective at protecting against infection with HPV strains 16 and 18 which might lead to cervical intraepithelial neoplasia (CIN) lesions and cervical cancer [8, 9], and they are available in China in 2016. 4-valent HPV vaccine is recommended for female aged 20–45 [10]. The acceptance of HPV vaccination will be expected to depend on factors such as knowledge of HPV infection and its link to cervical cancer [11].

However, levels of HPV awareness among the general population are poor throughout the world. Systematic review of studies from several countries suggests that only 15–31% of respondents have even heard of HPV, regardless of age, while only 0.6–11% know that HPV is a risk factor for cervical cancer [12]. Surveys in China paint a similar picture: Among the general population of Chinese women, 24% have heard of HPV [13]. In a cross-sectional survey on parents in Jinan, China, 22.63% have heard of HPV, 40.8% parents are willing to accept HPV vaccination for children [14]. Only 10% of high school students have heard of HPV, and only 19% know that HPV infection can lead to cervical cancer [15]. Among junior middle school students in Jinan, China, 15.5% have heard of HPV, and 66.9% are willing to receive the HPV vaccination [16].

It is unclear how much knowledge the general population in western China has about HPV, such as how the virus is transmitted, how infection can be detected, and whether it is linked to cervical cancer. Therefore, we wanted to examine in detail the HPV knowledge of women and their attitude to HPV vaccination. The results of this study may help guide the design and improvement of interventions and campaigns to sensitize women to HPV infection and vaccination, thereby helping reduce incidence of cervical cancer.

## Methods

### Study design, sample selection and data collection

All participants provide informed written consent to take part in our study. Healthy women undergoing routine physical examinations in the Health Management Center of West China Hospital of Sichuan University between January and December 2013 were invited to take a single survey. To be enrolled in the study, women had to be able to read the questionnaire on their own and understand it on their own or with the help of staff, they were given the paper-based questionnaire to complete by themselves and then return to staff. Women were not eligible if they were older than 75 or had a history of cervical cancer.

The target study population was 1300 women, which assuming a 75% response rate should provide a sample adequate for estimating proportions for which the 95% confidence interval would have a maximum width of 10%. This corresponds to maximum error of ±5% for all estimates of proportions.

### Survey instrument

A self-administered questionnaire was designed specifically for the present study and based on the literature [17–19]. The questionnaire (as shown in Additional files 1 and 2) included outcome variables and correlate variables, and it omitted questions about age at menarche and the menstrual cycle in order to reduce bias due to cognitive boredom and fatigue. The questionnaire was piloted with a group of 30 women not included in the final study population in order to assess ease of completion. During pilot testing, several women asked whether HPV is related to breast cancer. This likely reflects the fact that the Chinese name of HPV includes the word “papilla”. To avoid such confusion in the full study, a statement that HPV has no relationship with breast cancer was inserted into the questionnaire. The survey was given to participants together with a cover letter explaining its purpose. Questionnaires were anonymous, ensuring confidentiality.

#### Outcome variables

Question 1 (Q1) asked respondents whether they had heard of HPV. Surveys on which the response to Q1 was “no” were excluded from analysis of responses to Q2–6. These five questions asked whether respondents knew various details about HPV infection. Q1–6 were as follows:

Q1. Have you heard of HPV? (*options*: yes, no).

Q2. Do you think HPV causes cervical cancer?(*options*: yes, no, don’t know).

Q3. Is HPV infection asymptomatic? (*options*: yes, no, don’t know).

Q4. Is HPV infection a sexually transmitted disease (STD)? (*options*: yes, no, don’t know).

Q5. Can HPV infection cause an abnormal Pap test? (*options*: yes, no, don’t know).

Q6. Did you know that human papillomavirus and human immunodeficiency virus (HIV) are different? (*options*: yes, no).

The final question, Q7, was analyzed for all respondents, regardless of whether they had heard of HPV or not.

Q7. Are you willing to receive the HPV vaccine which can protect against HPV infection? (*options*: yes, no).

#### Correlate variables

The survey also collected data on respondents’ age, ethnicity, educational background, health, income, relationship status, frequency of sexual intercourse, use of condoms, Pap testing, family history of cancer, and history of penis or prostate cancer in the husband/partner. These data were used to explore factors associated with levels of HPV knowledge.

### Statistical analysis

Data were managed in EXCEL and analyzed using SPSS 17.0 (IBM, Chicago, IL). Descriptive statistics were used to evaluate knowledge about HPV infection and vaccine acceptance. Responses of “don’t know” were re-coded as “no” in the statistical analyses. Associations between respondent characteristics and level of knowledge or vaccine acceptance were explored in univariate analysis using the chi-squared test. Factors in the univariate analysis for which the associated *P* < 0.05 were then used in multivariate logistic regression.

## Results

### Characteristics of respondents

A total of 1300 questionnaires were distributed and 1260 questionnaires were recovered, corresponding to a recovery rate of 96.92%. Of the returned questionnaires, 151 were excluded because they were incomplete. The remaining 1109 questionnaires were accepted as valid and used in the final analysis, corresponding to an effective response rate of 88.02% (1109/1260).

The median age among all respondents was 40 (range, 18–76), and the mean age was 40.42 ± 9.02 (Table 1). Nearly all respondents (97.9%, 1086/1109) were Han Chinese. Most had at least a bachelor’s degree, an annual income above 70,000 RMB (approximately 10,700 USD), and were married or cohabiting. Most respondents reported having taken a Pap test at least once (75%), being in very good health (65%) and never having had an STI (98%).

**Table 1:** Demographic characteristics of respondents (*n* = 1109) and univariate analysis of HPV knowledge and HPV vaccine acceptance

Variable	All women	Heard of HPV		Knew HPV causes cervical cancer		Willing to take HPV vaccine	
	*N* (%)	*N* (%)	*p*	*N* (%)	*p*	*N* (%)	*p*
Age, yr							
18–29	135 (12.2)	45 (33.3)	0.039	19 (14.1)	0.147	85 (63.0)	0.002
30–64	955 (86.1)	274 (28.7)		152 (15.9)		478 (50.1)	
65–75	19 (1.7)	1 (5.3)		0		5 (26.3)	
Ethnicity							
Han	1086 (97.9)	314 (28.9)	0.767	170 (15.7)	0.137	552 (50.8)	0.075
Minority	23 (2.1)	6 (26.1)		1 (4.3)		16 (69.6)	
Education							
Illiterate	19 (1.7)	4 (21.1)	0.00	1 (5.3)	0.001	11 (57.9)	0.772
Pre-high school	132 (11.9)	14 (10.6)		9 (6.8)		71 (53.8)	
High school	74 (6.7)	26 (35.1)		19 (25.7)		36 (48.6)	
Bachelor’s	716 (64.6)	241 (33.7)		122 (17.0)		359 (50.1)	
Postgraduate	168 (15.1)	35 (20.8)		20 (11.9)		91 (54.2)	
Family annual income, RMB							
< 40,000	248 (22.4)	59 (23.8)	0.050	34 (13.7)	0.200	116 (46.8)	0.259
40,000-70,000	367 (33.1)	123 (33.5)		67 (18.3)		185 (50.4)	
70,000-120,000	236 (21.3)	62 (26.3)		29 (12.3)		131 (55.5)	
> 120,000	258 (23.3)	76 (29.5)		41 (15.9)		136 (52.7)	
Marital status							
Married/cohabiting	1083 (97.7)	313 (28.9)	0.826	167 (15.4)	0.996	560 (51.7)	0.035
Single	26 (2.3)	7 (26.9)		4 (15.4)		8 (30.8)	
Frequency of sexual intercourse, per week							
< 2 times	775 (69.9)	221 (28.5)	0.648	124 (16.0)	0.492	385 (49.7)	0.139
2–7 times	315 (28.4)	95 (30.2)		43 (13.7)		170 (54.0)	
> 7 times	19 (1.7)	4 (21.1)		4 (21.1)		13 (68.4)	
History of STI							
Yes	28 (2.5)	10 (35.7)	0.417	7 (25.0)	0.155	552 (51.1)	0.525
No	1081 (97.5)	310 (28.7)		164 (15.2)		16 (57.1)	
Condom use							
Yes	506 (45.6)	165 (32.6)	0.011	83 (16.4)	0.406	253 (50.0)	0.458
No	603 (54.4)	155 (25.7)		88 (14.6)		315 (52.2)	
Health status							
Poor	24 (2.2)	8 (33.3)	0.711	5 (20.8)	0.300	9 (37.5)	0.394
Not very good	357 (32.2)	98 (27.5)		47 (13.2)		183 (51.3)	
Very good	728 (65.6)	214 (29.4)		119 (16.3)		376 (51.6)	
Family history of cervical cancer							
Yes	8 (0.7)	6 (75.0)	0.004	4 (50.0)	0.007	7 (87.5)	0.039
No	1101 (99.3)	314 (28.5)		167 (15.2)		561 (51.0)	
Family history of non-cervical cancer							
Yes	147 (13.3)	55 (37.4)	0.014	27 (18.4)	0.288	89 (60.5)	0.015
No	962 (86.7)	265 (27.5)		144 (15.0)		479 (49.8)	
Partner has penis or prostate cancer							
Yes	11 (1.0)	3 (27.3)	1.00	0 (0.0)	0.155	4 (36.4)	0.322
No	1098 (99.0)	317 (28.9)		171 (15.6)		564 (51.4)	
Pap testing							
Never	284 (25.6)	62 (21.8)	0.003	32 (11.3)	0.006	148 (52.1)	0.444
Uncertain	360 (32.5)	102 (28.3)		49 (13.6)		192 (53.3)	
Regular	465 (41.9)	156 (33.5)		90 (19.4)		228 (49.0)	

### Knowledge about HPV and HPV infection

Knowledge about HPV and HPV infection was poor among our respondents. Only 28.85% (320/1109) had ever heard of it. Among this subgroup of 320 women who had heard of the virus, 23.75% (76/320) knew that HPV infection is asymptomatic, 49.38% (158/320) knew that HPV infection is an STD, 47.81% (153/320) knew that HPV causes abnormal Pap test results, and 28.75% (92/320) knew that HPV is different from HIV. In this subgroup, 8.13% (26/320) correctly answered Q2–6 (Table 2).

**Table 2:** HPV knowledge and HPV vaccine acceptance among respondents

Question	*N*	%
Q1. Have you heard of HPV?		
Yes	320	28.85
No	789	71.15
Q2. Do you think HPV causes cervical cancer?^a^		
Yes	171	53.44
No/ don’t know	149	46.56
Q3. Is HPV infection asymptomatic?^a^		
Yes	76	23.75
No/ don’t know	244	76.25
Q4. Is HPV infection a sexually transmitted disease (STD)?^a^		
Yes	158	49.38
No/ don’t know	162	50.62
Q5. Can HPV infection cause abnormal Pap test results?^a^		
Yes	153	47.81
No/ don’t know	167	52.19
Q6. Did you know that human papillomavirus and HIV are different?^a^		
Yes	92	28.75
No	228	71.25
Q7. Are you willing to receive the HPV vaccine, which can protect against HPV infection?		
Yes	568	51.22
No	541	48.78

^a^Among women who had heard of HPV, i.e. who responded ‘yes’ to Q1

Univariate analysis (Table 1) showed that women who had heard of HPV were more likely than other women to be younger than 65, be able to read, have a relatively high salary, have a family history of cancer, undergo Pap testing regularly and use condoms during intercourse. Multivariate analysis was performed while controlling for the following variables: age, educational background, family annual income, condom use, family history of cervical cancer, family history of non-cervical cancer, and Pap testing. This analysis identified the following factors as positively correlated with knowledge about HPV and HPV infection (Table 3): family history of any cancer, regular Pap testing and an educational background of high school or above.

**Table 3:** Mutivariate logistic regression to identify factors associated with HPV knowledge and HPV vaccine acceptance

Variable	Heard of HPV		Knew HPV causes cervical cancer		Willing to take HPV vaccine	
	OR	95% CI	OR	95% CI	OR	95% CI
Age, yr						
18–29	5.992	0.754–47.617			4.400	1.482–13.065
30–64	5.273	0.684–40.632			2.494	0.883–7.045
65–75	1.00				1.00	
Education						
Illiterate	1.170	0.356–3.844	0.476	0.060–3.781		
Pre-high school	0.446	0.225–0.883	0.562	0.246–1.287		
High school	1.952	1.031–3.695	2.374	1.162–4.852		
Bachelor’s	1.809	1.184–2.765	1.442	0.862–2.414		
Postgraduate	1.00		1.00			
Family annual income, RMB						
< 40,000	1.00					
40,000-70,000	1.380	0.941–2.024				
70,000-120,000	0.866	0.562–1.335				
> 120,000	0.940	0.617–1.433				
Marital status						
Married/cohabiting					2.210	0.939–5.203
Single					1.00	
Condom use						
Yes	1.150	0.868–1.525				
No	1.00					
Family history of cervical cancer						
Yes	8.293	1.609–42.732	6.039	1.457–25.026	6.708	0.819–54.945
No	1.00		1.00		1.00	
Family history of non-cervical cancer						
Yes	1.569	1.076–2.287			1.572	1.099–2.248
No	1.00				1.00	
Pap testing						
Never	0.689	0.477–0.994	0.629	0.404–0.980		
Uncertain	0.928	0.677–1.272	0.755	0.511–1.115		
Regular	1.00		1.00			

### Knowledge that HPV causes cervical cancer

Among women who had heard of HPV, 53.44% (171/320) knew that HPV causes cervical cancer. Univariate analysis showed that these women were more likely than other women to be able to read, to undergo Pap testing regularly and to have a family history of cervical cancer (Table 1). Multivariate analysis was performed while controlling for the following variables: educational background, family history of cervical cancer, and Pap testing. The results identified the following factors as positively correlated with knowledge that HPV causes cervical cancer (Table 3): regular Pap testing, family history of cervical cancer and an educational background of high school or above.

### Willingness to take the HPV vaccine

Among all respondents, including those had heard of HPV or not, 51.22% reported being willing to take the HPV vaccine. This willingness may reflect knowledge acquired before the survey and/or knowledge gained upon reading the survey question, which explicitly mentioned that the vaccine can protect against cervical cancer. Univariate analysis showed that these women were more likely to be younger than 65, to be married or cohabiting, and to have a family history of cancer (Table 1). Multivariate analysis was performed while controlling for age, relationship status, and family history of cervical or non-cervical cancer. The results identified the following factors as positively correlated with willingness to take the HPV vaccine (Table 3): age 18–29 and family history of non-cervical cancer.

## Discussion

This study provides the first insights into HPV awareness and attitudes towards the HPV vaccine among women in western China. Only 29% of our respondents had heard of HPV, and few knew that it is associated with cervical cancer. Women with a family history of cervical cancer, women who regularly underwent Pap testing and women with at least a high school education were more likely to have greater knowledge about HPV. Despite the low level of knowledge about HPV and HPV infection, half of respondents (51%) reported being willing to take the HPV vaccine. These results suggest the urgent need to disseminate knowledge about HPV and its association with cervical cancer, and they further suggest that such campaigns are likely to be effective at increasing willingness to be vaccinated.

The level of HPV knowledge among our respondents is lower than that reported in a 2010 survey of wealthier women in Australia [20], 63% of whom knew what HPV was. At the same time, the awareness level in our study is higher than that reported in Africa [21], suggesting that women in richer countries may know more about HPV. The potential relationship between socioeconomics and HPV knowledge requires further study, since published study populations show a range of cultural, socioeconomic and other differences. In 2012, a multi-center epidemiological survey of women aged 15–59 in China found that only 24% had heard of HPV [13]. This discrepancy from our results may reflect the lower literacy rate among the women in that study. Our observation that annual income was not significantly associated with HPV knowledge in our study population may reflect that most women in our sample were economically secure and had completed university. These women have likely been exposed to information about HPV to a greater extent than the general public.

Our results identified several factors that positively correlated with HPV knowledge: family history of any cancer, regular Pap testing and an educational background of high school or above. HPV knowledge was not, however, associated with age, ethnicity, health status, family annual income, relationship status, frequency of sexual relations, history of STDs, or condom use. Our results suggest that HPV awareness depends more on education and exposure to HPV or cancer than on family income. One implication of our findings is that HPV awareness campaigns should be targeted at groups that normally receive little or no education about health in general or cancer in particular, including poorer, less educated groups without access to advanced healthcare facilities.

Such education campaigns should aim not merely to raise awareness of HPV but also to emphasize its link with cervical cancer. Most women in our sample who had heard of HPV did not know that the virus causes cervical cancer. Women who had a higher educational background or a family history of cervical cancer or who regularly underwent Pap testing were more likely to know about this link. Education programs about HPV have been shown to be effective at improving awareness of cervical cancer, the association between HPV and cervical cancer and perception of HPV vaccines [22, 23]. Studies in China and other countries suggest that healthcare providers often lack HPV knowledge, which in part reflects the fact that DNA-based HPV tests have only recently arrived in the clinic [13, 24–26]. Given that women prefer to obtain health information from trusted sources, including physicians, health workers and audiovisual media [27], campaigns to raise awareness of HPV and its link to cervical cancer should target healthcare professionals as well as the general population.

Strikingly, our study found that despite quite low rates of knowledge about HPV and its association with cervical cancer, half of respondents were willing to take the HPV vaccine once they knew that the HPV vaccine can protect against HPV infection. The reason for this high willingness may be the rising incidence of cancer in China [2]. Interestingly, willingness to take the HPV vaccine did not depend on educational background or family annual income; instead, it depended on age and family history of cervical cancer. Women aged 18–29 and women with a family history of cervical cancer were more likely to be willing to take the vaccine. Other studies have also reported that younger people have more positive attitudes towards the HPV vaccine than older individuals [28–31]. Future work should explore reasons why women report that they are unwilling to take the vaccine.

The results of this survey should be interpreted with caution given that the format, wording of questions and sequence of questions can bias responses [32, 33]. In addition, our respondents were women who went to the Health Management Center of our university hospital for a physical examination, so they are likely to be more educated and financially better-off than the general population. Third, only a handful of respondents were non-Han, limiting our ability to assess effects of ethnicity. Previous work showed that HPV awareness can vary among ethnic groups [34], so this question should be pursued further. Fourth, our questionnaire did not take into account possible adverse effects and price of HPV vaccine in the survey, we might overestimate the willingness to take the HPV vaccine. Fifth, the multiple-choice format of the questionnaire increases the risk that our results overestimate HPV awareness in women in West China.

Despite these limitations, our results may be more reliable than some other studies because we did not analyze the responses of several questions for women who had not heard of HPV; as a result, our analysis of those questions should more accurately reflect knowledge among women previously exposed to HPV information. In addition, we added a statement that HPV had no relationship with breast cancer, a misconception that occurred in the pilot study because the Chinese name of HPV includes the word “papilla”.

## Conclusion

Our results point to an urgent need for education and intervention to raise awareness of HPV and its link to cervical cancer. At the same time, they provide encouragement that such education can increase willingness to take the HPV vaccine, since even in the absence of prior knowledge about HPV. Future work should examine the effects of such education on the psychology and health-seeking behaviors of women, and ultimately on the incidence of cervical cancer.

## Additional files

Additional file 1: Questionnaire (Chinese). (DOCX 17 kb)

Additional file 2: Questionnaire (English). (DOCX 18 kb)

## Abbreviations

CIN: Cervical intraepithelial neoplasia
DNA: Deoxyribonucleic acid
HIV: Human immunodeficiency virus
HPV: Human papilloma virus
STD: Sexually transmitted disease
