# Supplementary material for: Knowledge of HPV and acceptability of HPV vaccine among women in western China: a cross-sectional survey
Source: BMC Womens Health. 2018 Jul 27;18:130. doi: 10.1186/s12905-018-0619-8 (PMC6063014; doi:10.1186/s12905-018-0619-8)
Supplement: Supplementary file 1 — Questionnaire (Chinese). (DOCX 17 kb) [file 12905_2018_619_MOESM1_ESM.docx]

**调查问卷**

**一）一般项目：**

年龄：---- 民族：----

二）**婚姻状况、性生活状况**

- 婚姻状况：1.已婚

2.离异/丧偶**但**有性伴侣

3.离异/丧偶**无**性伴侣

- 性生活频率：1.<=1次/周 2.2-7次/周 3.>7次/周
- 常规避孕方式：1.避孕套 2.其他

三）**个人、配偶及家族健康状况：**

- 您平素健康状况 ：1.良 2.中 3.差
- 您现在或过去是否患性传播疾病？

1.是 2.否

- 您多长时间做一次宫颈细胞学检查？

1.从不 2.不定期 3.定期

- 家族史：患有宫颈癌病史（是 / 否 ）

患宫颈癌以外的其他癌症病史（是 / 否 ）

- 配偶有阴茎癌/前列腺癌病史 （是 / 否 ）

**四）文化程度**：1. 无文化 2．高中以下 3．高中

4．大学本科 5.>=研究生

**五）家庭收入:** 1. <4万/年 2. 4-7万/年

3. 7-12万 4. 12万以上/年

**二、HPV感染相关的知识**

1、 您是否听说过人乳头瘤病毒(HPV)？

1)是 2）否

2. HPV感染会导致宫颈癌吗？

1）会 2）不会 3）不知道

3. 感染HPV后是否有症状？

1）有 2）没有 3）不知道

4. HPV感染的主要途径是性行为？

1）是 2）不是 3）不知道

5. HPV感染会引起宫颈细胞学异常

1）是 2）不是 3）不知道

6. 人乳头瘤病毒与HIV是不同的？

1）是 2）不是

7. 若有通过抗HPV感染预防宫颈癌的疫苗在中国上市，您是否愿意接种呢？

1）愿意 2）不愿意
