# Supplementary material for: Knowledge of HPV and acceptability of HPV vaccine among women in western China: a cross-sectional survey
Source: BMC Womens Health. 2018 Jul 27;18:130. doi: 10.1186/s12905-018-0619-8 (PMC6063014; doi:10.1186/s12905-018-0619-8)
Supplement: Supplementary file 2 — Questionnaire (English). (DOCX 18 kb) [file 12905_2018_619_MOESM2_ESM.docx]

Part I

1. **General items:**

Age:______ Ethnicity：______

1. **Marital status and sexual status**

●Marital status: 1. Married/cohabiting 2. Single

●Frequency of sexual intercourse, per week: 1. <2 times; 2. 2-7 times; 3. >7 times

●Routine contraception 1. Condom 2. Other ways

**3）Personal, spouse and family health status:**

●Your Health status? 1. Poor 2. Not very good 3. Very good

●Whether have sexually transmitted disease ? 1.Yes 2. No

●Frequency of Pap testing? 1. Never 2. Uncertain 3. Regular

●Family history of non-cervical cancer 1.Yes 2. No

●Family history of non-cervical cancer 1.Yes 2. No

●Partner has penis or prostate cancer 1.Yes 2. No

**4) Education background**  1. Illiterate 2. Pre-high school

3. High school 4. Bachelor’s 5. Postgraduate

|  |
| --- |
|  |
|  |
|  |

**5) Family annual income, RMB** 1. <40,000 2. 40,000-70,000

3. 70,000-120,000 4. >120,000

|  |
| --- |
|  |
|  |
|  |

Part II

**HPV infection-related knowledge questionnaire**

Q1. Have you heard of HPV?

1.yes 2.no

Q2. Do you think HPV causes cervical cancer？

1.Yes 2.no 3. don’t know

Q3. Is HPV infection asymptomatic?

1.yes 2. no 3. don’t know

Q4. Is HPV infection a sexually transmitted disease (STD)?

1.yes 2. no 3. don’t know

Q5. Can HPV infection cause an abnormal Pap test?

1.yes 2. no 3. don’t know

Q6. Did you know that human papillomavirus and human immunodeficiency virus(HIV) are different?

1.yes 2. no

Q7. Are you willing to receive the HPV vaccine which can protect against HPV infection?

1.yes 2. no
